# Metabolomic analysis and mass spectrometry imaging after neonatal stroke and cell therapies in mouse brains

**DOI:** 10.1038/s41598-020-78930-x

**Published:** 2020-12-14

**Authors:** Emi Tanaka, Yuko Ogawa, Ritsuko Fujii, Tomomi Shimonaka, Yoshiaki Sato, Takashi Hamazaki, Tokiko Nagamura-Inoue, Haruo Shintaku, Masahiro Tsuji

**Affiliations:** 1 Department of Pediatrics, Osaka City University Graduate School of Medicine, Osaka, Japan; 2 Institute of Biomedical Research and Innovation, Kobe, Japan; 3 Division of Bioenergetics, Research Center for Artificial Photosynthesis, Osaka City University, Osaka, Japan; 4 Division of Molecular Materials Science, Graduate School of Science, Osaka City University, Osaka, Japan; 5 Analysis Division, Research Center for Artificial Photosynthesis, Osaka City University, Osaka, Japan; 6 Division of Neonatology, Center for Maternal-Neonatal Care, Nagoya University Hospital, Nagoya, Japan; 7 Department of Cell Processing and Transfusion, Institute of Medical Science, The University of Tokyo, Tokyo, Japan; 8 Department of Food and Nutrition, Kyoto Women’s University, 35 Kitahiyoshi-cho, Imakumano, Higashiyama-ku, Kyoto, 605-8501 Japan; 9 Department of Regenerative Medicine and Tissue Engineering, National Cerebral and Cardiovascular Center, Suita, Japan

**Keywords:** Neuroscience, Stem cells in the nervous system, Disease model, Stem cells, Haematopoietic stem cells, Mesenchymal stem cells, Mass spectrometry, Metabolomics, Imaging

## Abstract

Ischemic brain injury provokes complex, time-dependent downstream pathways that ultimately lead to cell death. We aimed to demonstrate the levels of a wide range of metabolites in brain lysates and their on-tissue distribution following neonatal stroke and cell therapies. Postnatal day 12 mice underwent middle cerebral artery occlusion (MCAO) and were administered 1 × 10^5^ cells after 48 h. Metabolomic analysis of the injured hemisphere demonstrated that a variety of amino acids were significantly increased and that tricarboxylic acid cycle intermediates and some related amino acids, such as glutamate, were decreased. With the exception of the changes in citric acid, neither mesenchymal stem/stromal cells nor CD34^+^ cells ameliorated these changes. On-tissue visualization with matrix-assisted laser desorption/ionization-mass spectrometry (MALDI-MS) imaging revealed that the signal intensity of glutamate was significantly decreased in the infarct area, consistent with the metabolomic analysis, while its intensity was significantly increased in the peri-infarct area after MCAO. Although cell therapies did not ameliorate the changes in metabolites in the infarct area, mesenchymal stem cells ameliorated the increased levels of glutamate and carnitine in the peri-infarct area. MALDI-MS imaging showed the location-specific effect of cell therapies even in this subacute setting after MCAO. These methodologies may be useful for further investigation of possible treatments for ischemic brain injury.

## Introduction

Neonatal encephalopathy is an important cause of mortality and cerebral palsy^1^. Neonatal stroke is one of the most common causes of neonatal encephalopathy, and there has been no proven treatment for it^2^. Recently, many researchers have focused on cell therapies because of their regenerative and neuroprotective effects. The effects on injured neonatal brains are thought to be multifactorial, involving regeneration, paracrine effects and inflammatory modulation. Our previous reports showed that both human umbilical cord blood CD34^+^ cell- and human umbilical-cord-derived mesenchymal stem/stromal cell (MSC)-based therapies applied 48 h after permanent middle cerebral artery occlusion (MCAO) had neuroprotective effects, which occurred through the modulation of the inflammatory reaction and the recovery of cerebral blood flow rather than by cell replacement at the injured site or cellular secretion of brain-derived neurotrophic factor (BDNF)^3,4^. Ischemic brain injury provokes complex, time-dependent downstream pathways that reach an inflammatory peak at approximately 24 h after injury. Regarding the timing of cell therapy, our previous studies have highlighted that cell therapies have a long therapeutic time window, while most studies have used the treatments within 24 h after injury. Here, we focused on the neuroprotective potential of two cell therapies, CD34^+^ cells and MSCs, which are the most promising for and closest to clinical application. We used a neonatal model of permanent MCAO that is reproducible and consistent in terms of the severity of brain injury^5^, and we administered the cells 48 h after injury.

In recent years, metabolomics has received a great deal of attention, and researchers have demonstrated changes in low-molecular-weight metabolites after ischemic brain injury or treatment by using mass spectrometry (MS) or magnetic resonance spectroscopy (MRS). Regarding neonatal brain injury, metabolic profiles after hypoxic-ischemic encephalopathy or asphyxia have been examined in both clinical cases^6,7^ and large animal models^8–10^, while few studies have investigated small animals. No study has applied metabolomics in neonatal stroke models or after cell therapies in any neonatal brain injury model. We hypothesized that there are some potential mechanisms other than the immunomodulation of high-molecular-weight substances, including cytokines or trophic factors, underlying cell therapy; therefore, we focused on a large range of low-molecular-weight metabolites that could explain the energy level, excitotoxicity and oxidative stress observed in this process. When we used neonatal rodent animal models, however, the required sample volume for MS was larger than the infarct volume in the mouse model of neonatal stroke. As a result, the tissue samples for MS need to include tissue from both infarct and non-infarct regions and lose spatial information. A remarkable new technology, MS imaging, enables the detection and visualization of metabolites present in tissue sections by direct ionization and detection^11,12^. MS imaging based on matrix-assisted laser desorption ionization (MALDI) has been commonly used among biochemical researchers because of direct ionization on the tissue sections without labeling^13^. As a detector, we used a Fourier transform ion cyclotron resonance (FT-ICR) mass analyzer, which has high mass resolution in a single measurement over a wide molecular weight range, to contrast with the holistic metabolomic analysis^14–17^. We targeted a mass range of *m*/*z* 71–1000, and succeeded in detecting glucose, carnitine, glutamate and glutathione simultaneously and visualized them in the neonatal mouse brain. The combination of metabolomic analysis and MS imaging may contribute to a better understanding of the changes that occur during ischemia and the mechanism of functional improvement following cell therapy.

The objective of this study was to investigate the changes in metabolites in the neonatal brain during the subacute phase after stroke and determine how CD34^+^ cell- and MSC-based therapies improve brain metabolism using two types of MS. First, we conducted a holistic metabolomic analysis of brain extracts 72 h after permanent MCAO. Second, we performed MALDI-MS imaging to assess some important metabolites on-tissue. The brain metabolic profile and the visualization of the distribution of its metabolites may provide a further understanding of the effects of cell therapy. We demonstrated the changes in metabolites following MCAO and the two cell therapies through metabolomic analysis of homogenized tissue samples and MS imaging of tissue sections, which shows location-specific information. These methods provide complementary results.

## Results

### Metabolomic responses to MCAO

**Figure 1:**
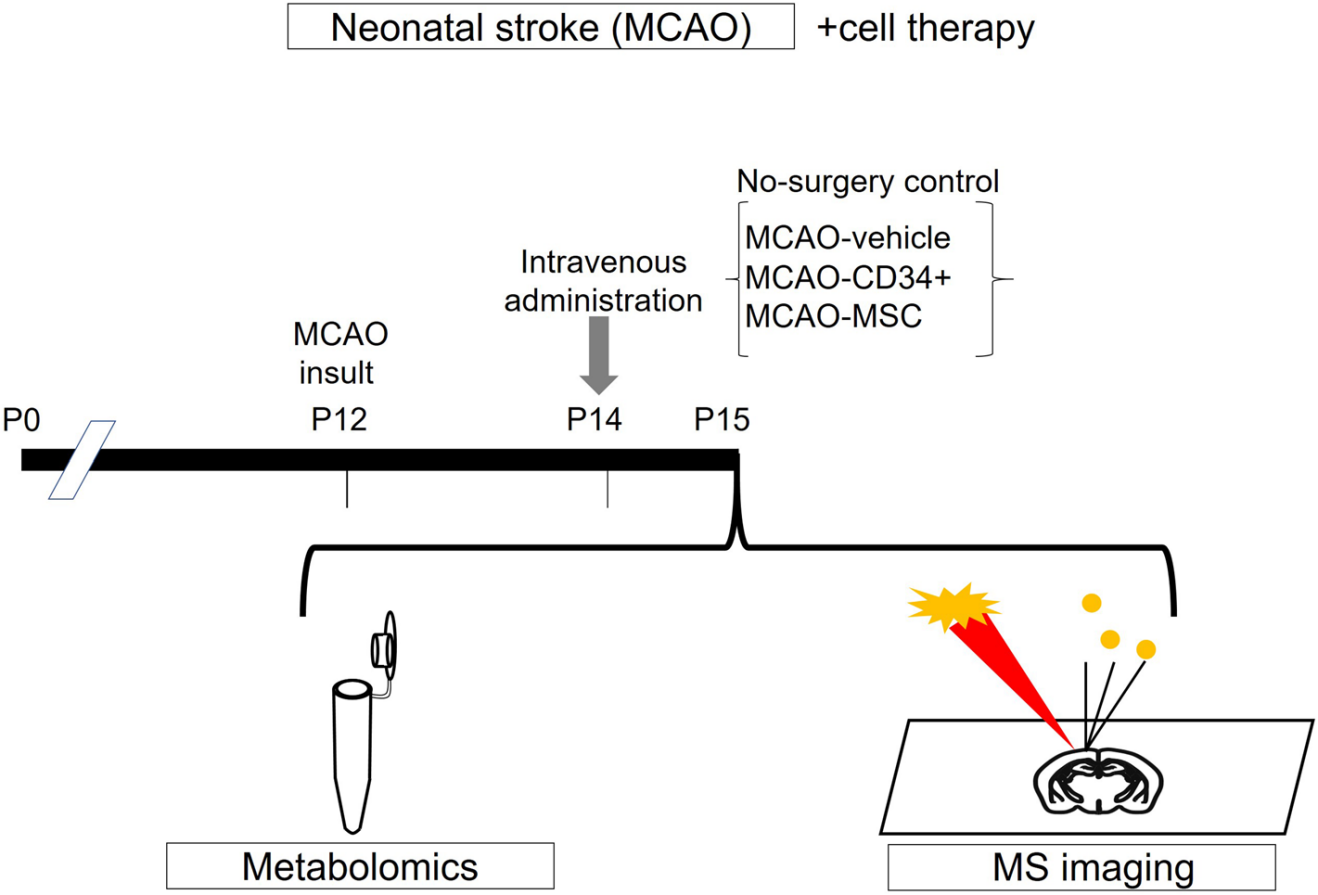
The experimental scheme. On postnatal day 12 (P12), mice underwent permanent middle cerebral artery occlusion (MCAO). Two days after the injury (P14), they were administered vehicle, umbilical-cord-blood-derived CD34^+^ cells (CD34^+^), or umbilical-cord-derived mesenchymal stem/stromal cells (MSCs) intravenously (i.v.). At P15, brain samples were collected. For metabolomic analysis, brain extracts from the injured hemispheres were analyzed by CE-TOF MS, and coronal brain sections from another cohort of mice were subjected to MS imaging.

In the present study, we performed a holistic evaluation of the metabolic profile after neonatal stroke, i.e., permanent MCAO, and evaluated the changes in this profile after cell therapy. Neonatal mice were divided into four groups: no-surgery control, MCAO-vehicle, MCAO-CD34^+^ cell, and MCAO-MSC (*n* = 5 in each group). Each sample, consisting of 20–30 mg of 2-mm-thick coronal sections of whole injured hemispheres, was obtained 72 h after MCAO, which was equivalent to 24 h after cell therapy (Fig. 1). We targeted 900 metabolites with capillary electrophoresis time-of-flight mass spectrometry (CE-TOF MS), and 151 metabolites were detected using the relative intensity per area. We used principal component analysis and heat map clustering, which provides an overview of all observed metabolites in the data.

**Figure 2:**
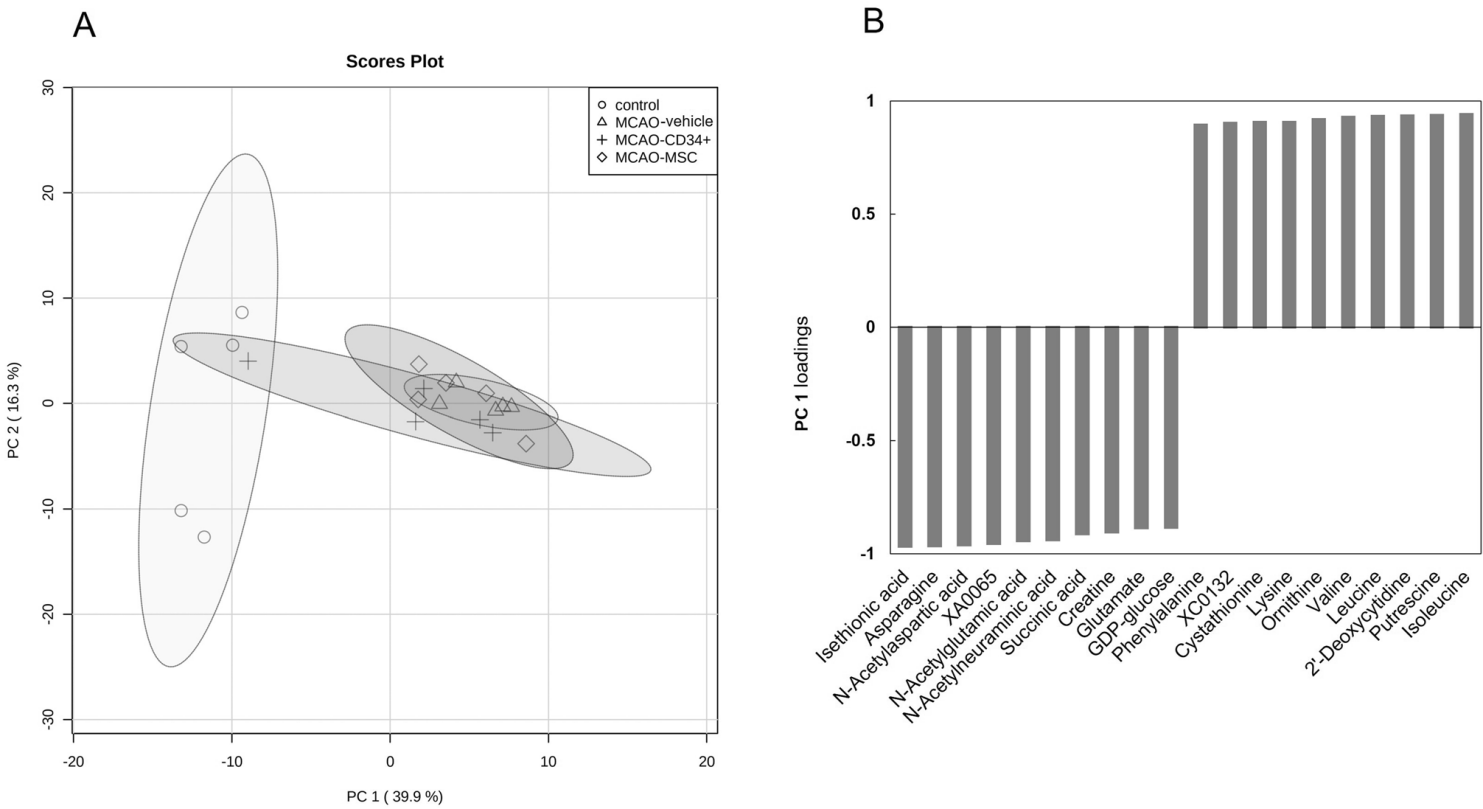
Principal component analysis of cerebral metabolomic changes 72 h after MCAO vs no surgery. CE-TOF MS data for 151 detected metabolites were subjected to principal component analysis after being split into four groups. (**A**) Score plot showing that the groups were well divided between the control (circle) and MCAO groups, excluding one outlier in the MCAO-CD34^+^ cell group (cross), according to the variation of principal component (PC) 1 (X-axis). (**B**) PC1 loading column plot of the top 20 metabolites. The listed metabolites strongly contributed to PC1, and vertical gray bars are distances in multivariate space between the average metabolite vectors for each group.

From the score plot of principal component analysis shown in Fig. 2A, it was apparent that the changes that occurred following MCAO compared with no surgery dominated principal component (PC) 1 variation on the X-axis, which describes 39.9% of the total variance in the data. However, the profile changes between the MCAO-vehicle and MCAO-cell-treated groups were not striking in PC1. Other components also did not discriminate the treatment groups. For PC1, the top 20 factor loadings are listed in Fig. 2B; they represent how each metabolite contributes to PC1 and are also considered coefficients of the correlation with the PC1 score. Fifty-eight out of 151 metabolites were significantly correlated with the PC1 score, with a strong correlation coefficient of > 0.7 (Suppl. 1), which indicates that the control and MCAO groups can be distinguished. Amino acids, such as isoleucine (Ile), leucine (Leu), valine (Val), lysine (Lys) and phenylalanine (Phe), were positively correlated with PC1 and were elevated after MCAO. A wide variety of essential amino acids, including branched-chain amino acids, were elevated as a possible result of protein degeneration and the reduced protein synthesis after MCAO. On the other hand, the levels of glutamate (Glu), asparagine (Asn), aspartate (Asp) and the Asp derivative *N*-acetylaspartate (NAA) were negatively correlated with PC1 scores, which means that they were decreased after MCAO. These molecules are widely present in neural cells. As Glu and NAA are the most abundant and second most abundant molecules, respectively, in the brain among these targeted metabolites, their decrease may be due to neuronal loss after MCAO. NAA is typically detected using NMR or MRS methods and is considered to be a metabolic marker for mitochondrial and neuronal function^18,19^. Moreover, the levels of TCA cycle intermediates, such as succinic acid, malic acid and fumaric acid, were also negatively correlated with the PC1 score. This suggested that energy generation was suppressed by MCAO.

**Figure 3:**
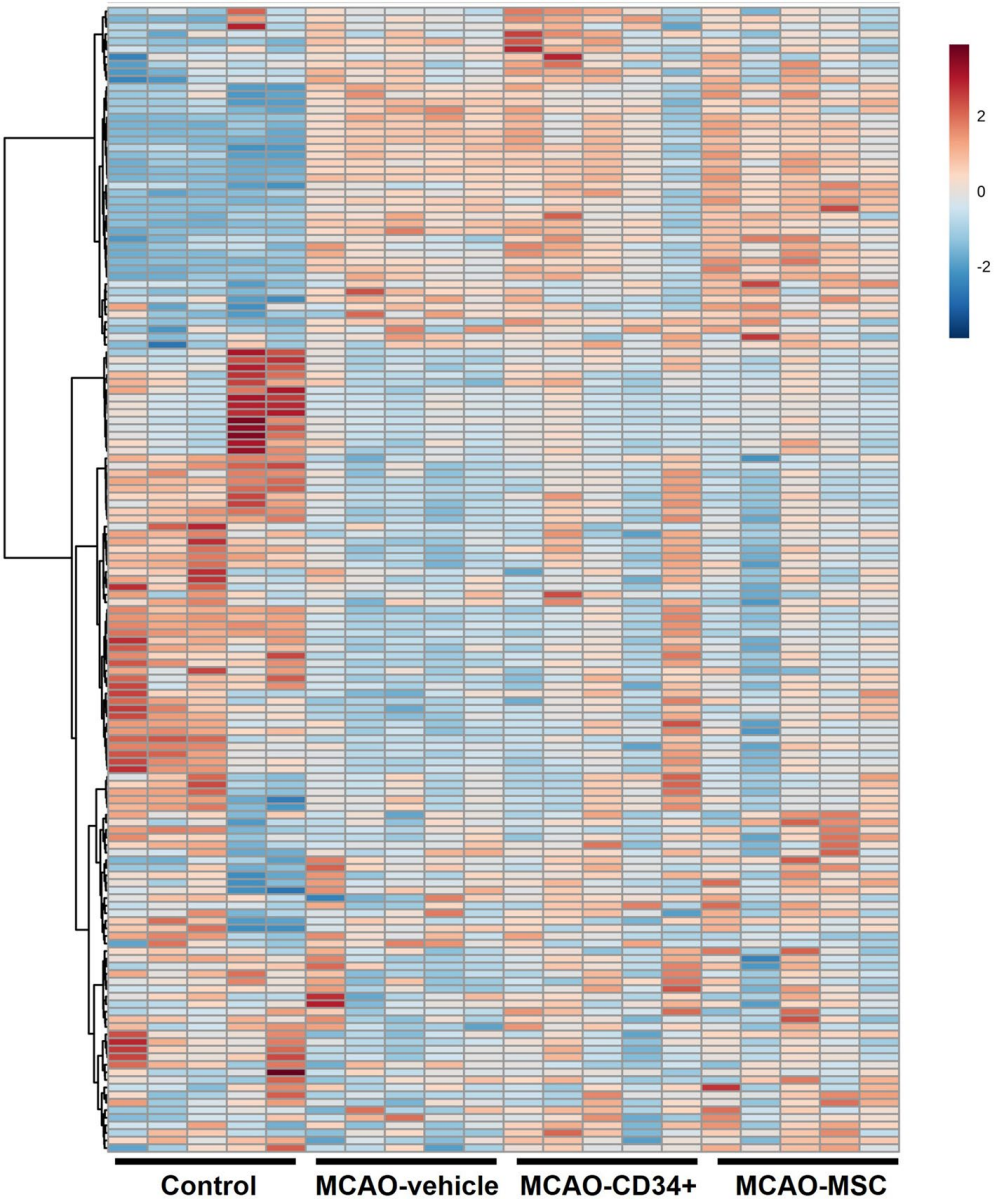
Heat map of all metabolites identified 72 h after MCAO. CE-TOF MS data from 151 metabolites detected in the injured hemisphere were visualized. The normalized mean values were displayed using MetaboAnalyst ver. 4 (https://www.metaboanalyst.ca/). Heatmap with labels are presented in Supplementary information 2.

The heat map analysis (Fig. 3 and Suppl. 2 with labels) shows the overview of all samples and metabolites at a glance. In addition to the observations from principal component analysis, the change between the control and MCAO groups was apparent in the heat map analysis.

### Metabolomic responses to cell treatment after MCAO

**Figure 4:**
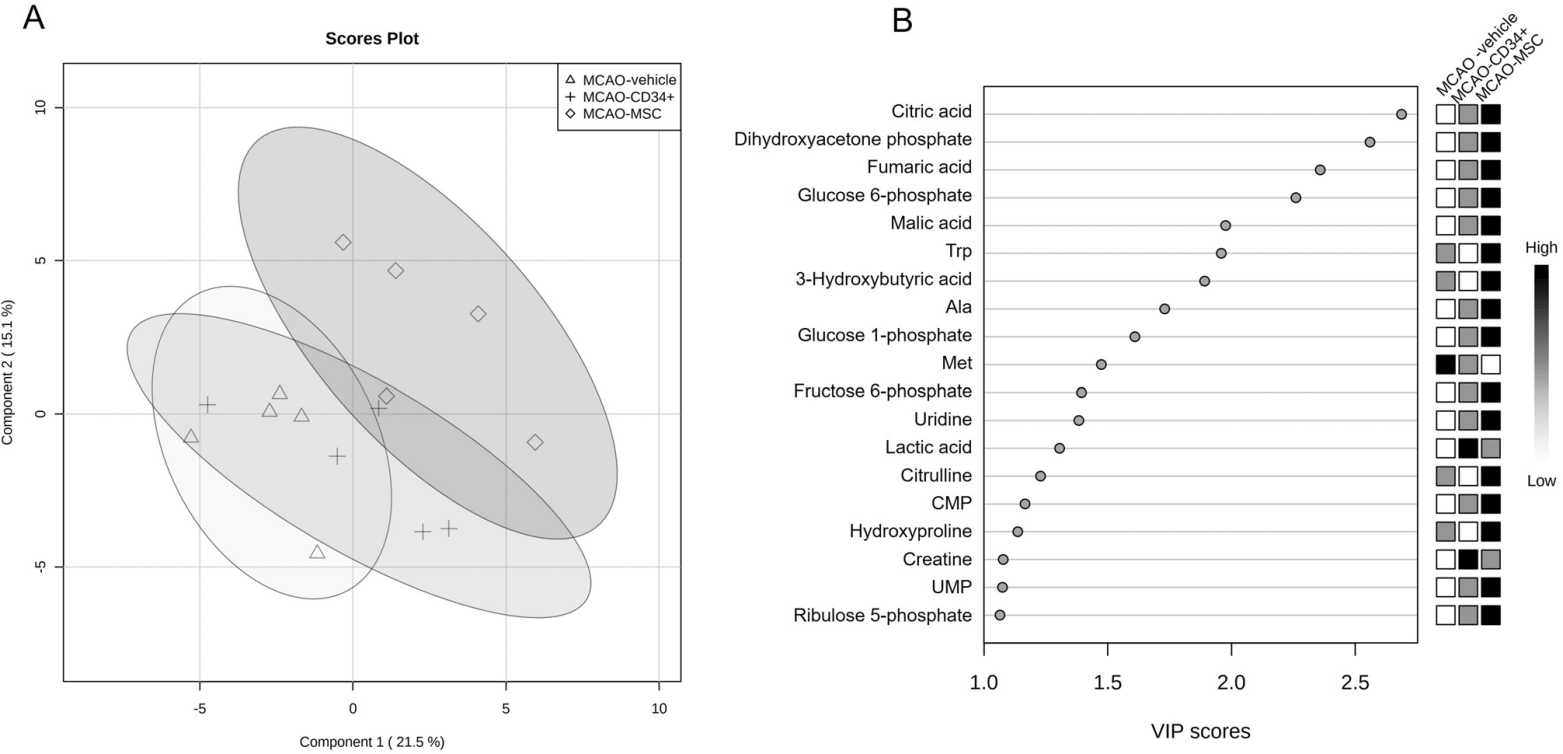
Multivariate analysis of cerebral metabolomic changes among the MCAO groups. Concentration data for 79 metabolites detected among the three MCAO groups were subjected to partial least-squares discriminant analysis (PLS-DA). The score plot illustrates the difference between the MCAO-vehicle group (triangle) and the MCAO-MSC group (diamond) (**A**). Variable importance in the projection (VIP) scores > 1 among the three MCAO groups were extracted and are shown in a score plot (**B**).

**Figure 5:**
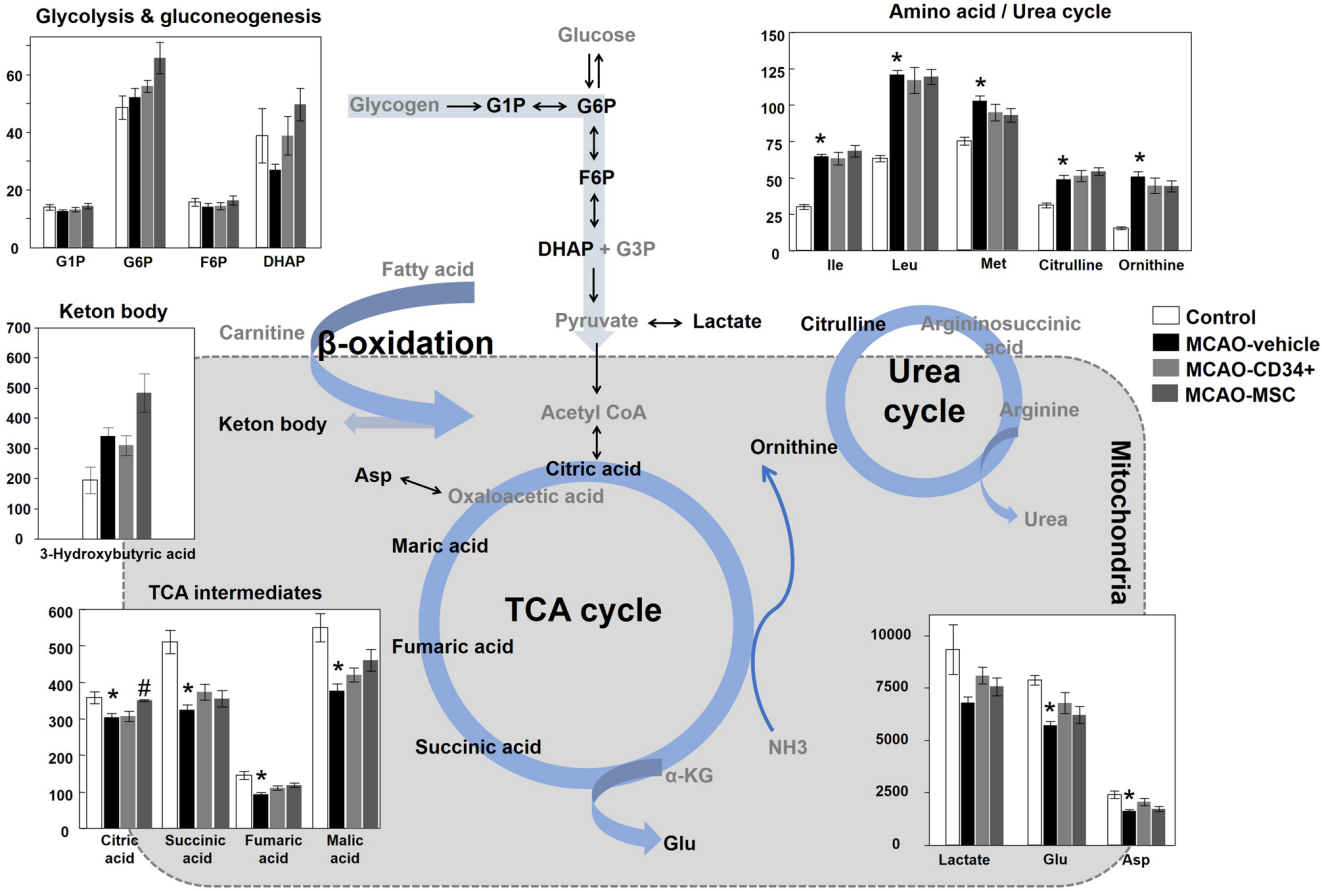
Central metabolic pathway map and comparative visualization of involved metabolites after MCAO. Differences in the brain levels of metabolites among the control, MCAO-vehicle, MCAO-CD34^+^ cell, and MCAO-MSC groups are presented in a bar graph. MCAO decreased TCA intermediates and neurotransmitter metabolites, while MCAO increased metabolites from the amino acid and urea cycles. Data indicate the mean ± the S.E.M. of five mice for each group. *p < 0.05 versus the control group. ^#^*p* < 0.05 versus the MCAO-vehicle group. G1P, glucose-1-phosphate; G6P, glucose-6-phosphate; F6P, fructose 6-phosphate; DHAP, dihydroxyacetone phosphate; G3P, glyceraldehyde 3-phosphate; Ile, isoleucine; Leu, leucine; Met, methionine; Glu, glutamate; Asp, aspartate; αKG, α-ketoglutaric acid; NH_3_, ammonia.

To investigate the effects of cell therapies, the concentrations of 79 metabolites were properly quantified from the aforementioned intensity analysis of 151 metabolites. Three groups (the MCAO-vehicle, MCAO-CD34^+^ cell, and MCAO-MSC groups) were reanalyzed using partial least-squares discriminant analysis (PLS-DA) by adding the grouping information. As shown in the PLS score plot (Fig. 4A), we could differentiate the MCAO-vehicle and MCAO-MSC groups in component 1. Metabolites that presented with variable importance in the projection (VIP) scores > 1 were extracted and are shown in Fig. 4B, which indicates the contributory variables in the PLS-DA model. Citric acid, fumaric acid and malic acid, TCA cycle intermediates, were highly ranked in the VIP scores. Dihydroxyacetone phosphate (DHAP), glucose 6-phosphate (G6P), glucose 1-phosphate (G1P), fructose 6-phosphate (F6P) and lactic acid, which are involved in glycolysis or gluconeogenesis, had VIP scores > 1. 3-Hydroxybutyric acid, known as a ketone body, was also highly important for distinguishing the groups. Figure 5 shows an overview of the metabolites in these critical pathways. In addition to the principal component analysis, the change between the control and MCAO groups was evident in some metabolites. Though no improvement in any metabolite except citric acid was observed after cell therapy, metabolomic analysis implied that intravenous cell administration altered some metabolic pathways in neonatal brains.

### Changes in the distribution of the metabolites after MCAO

**Figure 6:**
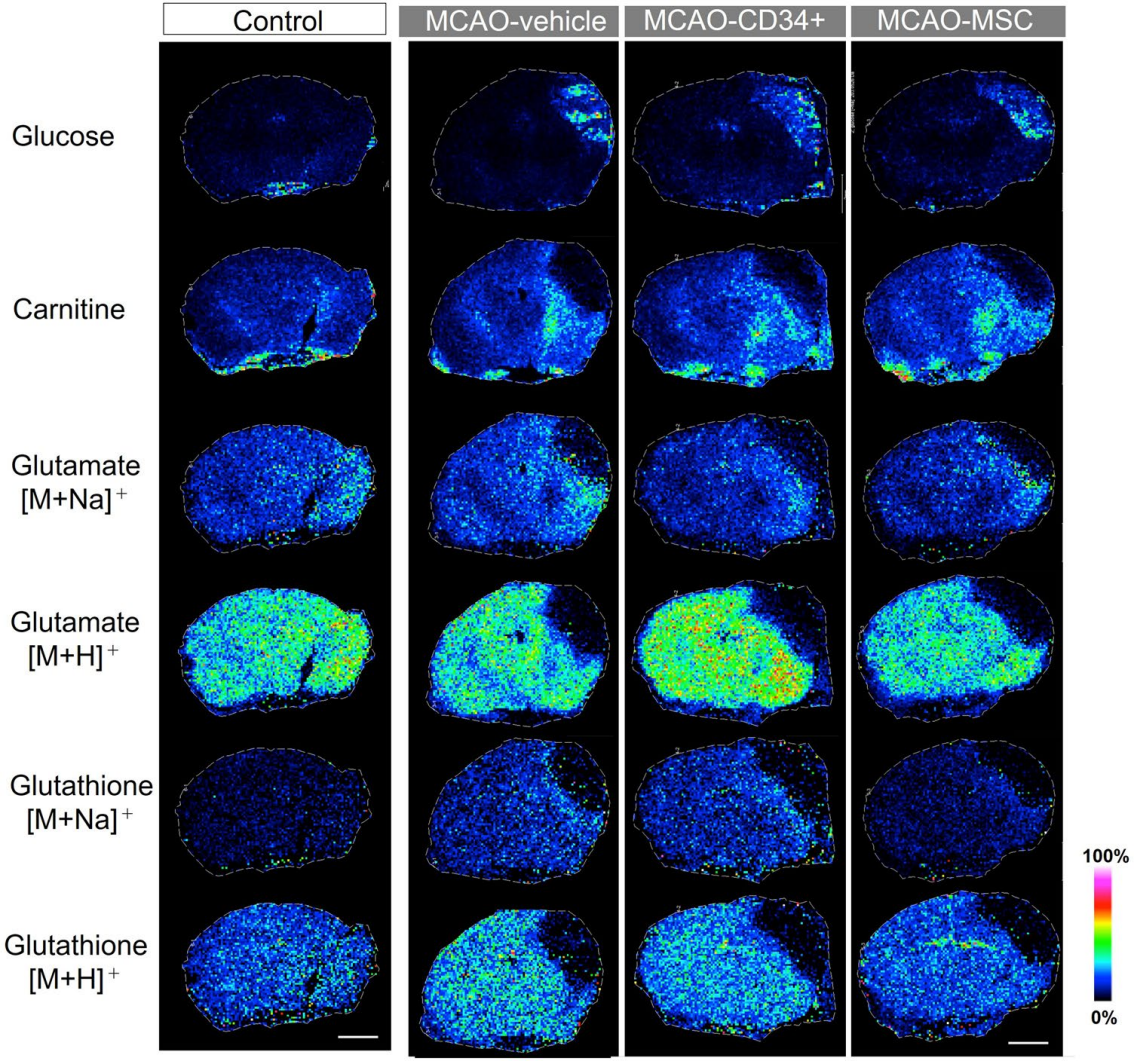
Mass spectrometry imaging. MS imaging was used to visualize the spatial changes and distributions of metabolites in the brains of mice that underwent MCAO. Metabolites were simultaneously visualized in a single scan, and the displayed brain images in each group were from a single mouse. Scale bars are 2.0 mm.

**Figure 7:**
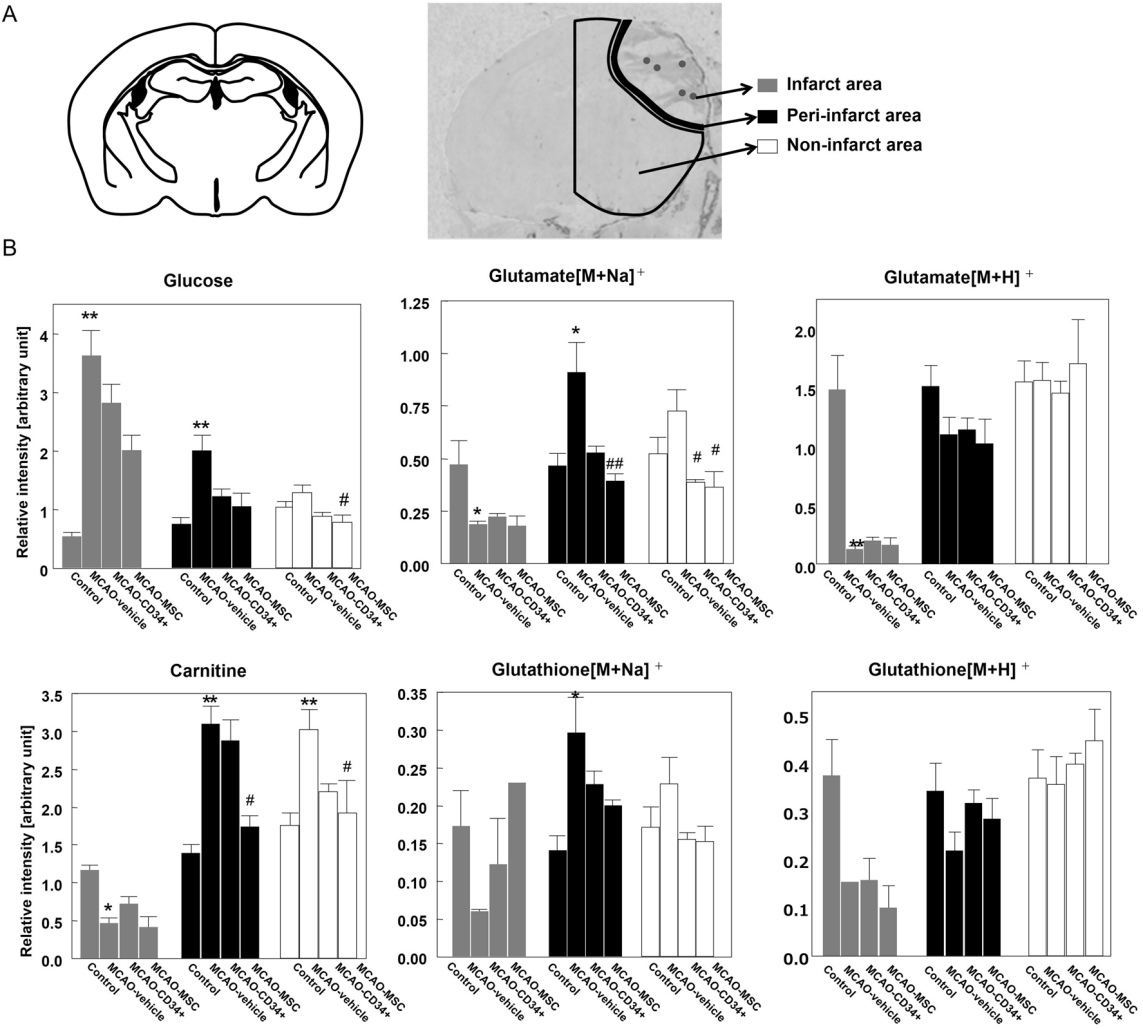
Comparative analysis of the metabolites oriented by area. After visualization of the metabolites by MS imaging, we compared their relative intensities in three separate areas: the ischemic area (average of five dots), the peri-infarct area, and the non-infarct area. (**A**) A schematic illustration representing the region of interest (ROI) that we used. As a control, we used the contralateral sides of brains from MCAO-vehicle mice. (**B**) The bar graph represents the average intensity of each ROI. Data are shown as the mean ± the S.E.M. of five mice for each group. * and ** indicate *p* < 0.05 and *p* < 0.01 versus the control group, respectively, and # and ## indicate *p* < 0.05 and *p* < 0.01 versus the MCAO-vehicle group, respectively.

To elucidate the spatial distribution of the metabolites, MALDI-MS imaging was performed for frozen brain sections 72 h after MCAO, which was the same time point used for the metabolomic analysis. We focused on 4 metabolites (glucose, carnitine, glutamate and glutathione) that could explain the energy status, excitotoxicity and redox status. These metabolites were ionized and detected using the same settings, such as matrix and ionization polarity. As shown in the mass spectra of the target molecules in Suppl. 3, both Na and H adducts were detected for glutamate and glutathione. After MCAO, all metabolites in the ischemic core except glucose had clearly decreased intensities (Fig. 6). Only glucose showed an increased intensity in the ischemic core, which may mean that functionally dead neurons could no longer use glucose for energy. Of note, carnitine and Na adducts of glutamate and glutathione had a relatively high distribution in the area surrounding the ischemic core. However, such slight differences are difficult to see in the qualitative images of Fig. 6 because the imaging software automatically optimizes the contrast focusing on the core in this MCAO model. To clarify these changes in relation to the ischemic lesion, we evaluated the regional intensities in three areas, e.g., the infarct area (ischemic core), the peri-infarct area, and the non-infarct area, and we used the contralateral hemisphere of vehicle-treated animals as a reference (Fig. 7A). The infarct area in this model was clearly demarcated 48 h after MCAO, and the peri-infarct area was defined as the 0.4 mm-thick border adjacent to the infarct area, which was based on our previous observation that microglial accumulation was reduced in the 0.4 mm-thick peri-infarct area after MSC therapy^4^. As Fig. 7B shows, glucose values in both the infarct area and the peri-infarct area were elevated after MCAO. On the other hand, the intensities of carnitine and both adducts of glutamate were significantly decreased in the infarct area, while the intensities of carnitine and glutamate [M + Na]^+^ in the peri-infarct area were significantly elevated after MCAO. The increased intensities of carnitine and glutamate [M + Na]^+^ were decreased significantly by MSC treatment and to a lesser extent by CD34^+^ cell treatment. On the other hand, the intensity of glutamate [M + H]^+^ did not show an increase in the peri-infarct area. Regarding glutathione, since there were nine or ten missing values for each adduct because of low intensities, no tendency was found except that the Na adduct had high intensity in the peri-infarct area. Overall, the cell therapies did not attenuate the levels of the metabolites in the infarct area; however, dramatic changes were observed, especially in the peri-infarct area.

## Discussion

In this study, we elucidated the metabolic changes that occur following neonatal MCAO using holistic analysis, and we visualized the distribution of glucose, carnitine glutamate and glutathione with MALDI-MS imaging. We also showed how two cell treatments (CD34^+^ cells and MSCs) affect the injured brain 72 h after MCAO. In the brain metabolic analysis, we could easily differentiate the control group from the MCAO groups. Most amino acids were significantly elevated, and TCA cycle intermediates were decreased together with some amino acids that are abundant in neural cells, such as glutamate, aspartate and asparagine. Although the effects of cell therapy were not clear in the metabolomic analysis, it was suggested that cell therapies, especially MSC therapy, affect the TCA cycle, glycolysis/gluconeogenesis and ketone body production. On-tissue visualization with MALDI-MS imaging revealed that neither treatment affected the intensities of metabolites measured in the infarct area, while MSCs restored the levels of carnitine and the Na adduct of glutamate in the peri-infarct area and the level of carnitine in the non-infarct area.

Many past studies in neonatal HIE models have focused on metabolic changes in the acute phase (until 24 h after an ischemic episode)^10,20–22^. As we believe the start of analysis at 48 h is more practical for decisions on cell administration in severe clinical settings, we have clarified the change in metabolites 24 h after the cell therapies, i.e., the subacute phase. At this phase, the levels of TCA cycle intermediates (succinic acid, fumaric acid, and malic acid) decreased, unlike the change during hypoxia. During ischemia, a lack of glucose and oxygen activates the anaerobic glycolysis pathway, which leads to an increased production of lactate within 1 min^23^. The TCA cycle is also affected, and some reports have shown the elevation of its intermediates as a result of dysfunction^10,24,25^. Chouchani et al. reported that the level of succinic acid increased during ischemia, and this increase returned to normal levels within 5 min following reoxygenation^24^. Subsequent reactive oxygen species (ROS) production leads to the corruption of the pyruvate dehydrogenase complex (PDHC), which results in the unavailability of pyruvate to form acetyl-CoA^26^. In addition, MALDI-MS imaging revealed that the infarct area resembled a defect, which means cell death. Our finding of elevated amino acid levels after MCAO was also a result of protein degeneration and a lack of protein synthesis. Therefore, we speculate that cell death and the unavailability of acetyl-CoA from glucose lead to a decrease in TCA intermediates. In the subacute phase, the clinically used parameter lactate was not associated with the change after MCAO, nor was ATP. As our MCAO model was reproducible and homogeneous, as shown in the principal component analysis of the MCAO-vehicle score plot, these metabolomic findings may accurately reflect the subacute changes in metabolites following neonatal stroke.

MS imaging revealed striking changes in the distributions of metabolites after MCAO. Glucose accumulated in the ischemic core, while carnitine, glutamate and glutathione concentrations were significantly decreased. Anaerobic glycolysis accelerates neuronal (and glial) glucose uptake by neurons during ischemia^27^. Our observed result of glucose accumulation in the infarct area and peri-infarct area is consistent with this fact. In addition, the amount of glucose accumulation was higher in the infarct area than in the peri-infarct area, suggesting that the glucose at the ischemic core was not utilized after uptake, presumably due to a loss of neuronal function or cell death. In the peri-infarct area, which we defined as the 0.4 mm-thick border adjacent to the infarct area, the levels of carnitine, Na adducts of glutamate and Na adducts of glutathione were significantly higher after MCAO. Hypoxia induces an imbalance in the Na/K ratio, which leads to cell death in the perfused area. Liu et al. revealed the distribution of Na^+^ and K^+^ following permanent MCAO in MS imaging, which showed that Na^+^ levels were higher in the infarct core^28^. On the other hand, they showed that glutamate levels were higher in the peri-infarct area and lower in the infarct core, although they did not discuss the change in the peri-infarct area. It is well known that the excitotoxic effect of excessive glutamate release following stroke causes acute and chronic neuropathologies. Therefore, we think that the higher distribution of glutamate [M + Na]^+^ in the peri-infarct area was more reasonable than the distribution of glutamate [M + H]^+^. Similarly, Bragin et al. reported that glutathione levels were higher in the penumbra after MCAO^29^. As glutathione has a critical function in maintaining cellular redox status and protecting against oxidative injuries, we also assume that the Na adduct of glutathione is preferable. Regarding carnitine, its distribution did not seem to be higher along the peri-infarct area. The definition of the peri-infarct area was based on the fact that microglial accumulation was reduced there after MSC therapy in our previous MCAO study^4^. As carnitine showed a higher distribution both in the peri-infarct area and non-infarct area, we speculate that the mechanism of accumulation is different from that observed for glutamate and glutathione. In a previous report investigating ischemic hearts, unique metabolic changes were also reported in the penumbra region, where higher energy charges and preferential entry of glucose metabolites into the TCA cycle may occur. Even in the acute phase after an ischemic episode, the penumbra reflects elevated levels of TCA cycle intermediates and glutamate^30^. The changes in the peri-infarct area may provide an alternative view of the penumbra and may be a potential target for treatment.

We focused on therapies with two types of cells, CD34^+^ cells and MSCs, which have the potential to improve metabolic changes after brain injury. Our group previously studied the phenotype after CD34^+^ cell or MSC therapy following neonatal MCAO. CD34^+^ cells improved cerebral blood flow and percent volume loss, while MSCs did not ameliorate volume loss but improved neurological behavior, partially due to reduced microglial accumulation, as indicated by histology^3,4,31^. Although these studies have not compared the two treatments in the same experimental design, both cell therapies following neonatal MCAO improved some of the phenotypes. We explored the changes after cell therapies from the perspective of low-molecular-weight metabolites using metabolomic analysis and MS imaging in this study. Although the change after cell therapy was not striking in metabolomic analysis, the PLS-DA model and VIP scores showed greater involvement of the TCA cycle, glycolysis/gluconeogenesis and ketone body production after MSC treatment than after CD34^+^ cell treatment. Additionally, MS imaging showed significant amelioration of the excessive accumulation of glutamate and carnitine after MSC treatment in the peri-infarct area. Glutamate is known as an excitotoxic neurotransmitter that is produced after hypoxia, ischemia and inflammation, and a higher glutamate concentration is associated with adverse outcomes^32–34^. MS imaging revealed that glutamate was highly localized to the peri-infarct area, where cell therapies could work effectively, even 72 h after MCAO. Carnitine transports long-chain fatty acids into the mitochondria for alternative energy production and for the normalization of excessive acylcarnitine levels. Regarding energy production, suckling infants have an increased ability to utilize fatty acids and ketone bodies to survive hypoglycemia^20^. The effective use of fatty acids and ketone bodies produces acetyl-CoA, which can be involved in the TCA cycle. In addition, ketone bodies and carnitine themselves may have neuroprotective effects against hypoxic brain injury^35–40^. This study demonstrated that continuous excitotoxicity and the lack of acetyl-CoA as an energy resource are improved by cell therapy, and we speculate that the effect on metabolites is better for MSC therapy than for CD34^+^ cell therapy.

There were some limitations of this study. First, as shown in the score plot of principal component analysis (Fig. 2A), one of the mice in the CD34^+^ group mice rather not belong to the injured groups. Although we cannot rule out the possibility of technical failure of MCAO insult for this pup, which we have never encountered in our previous experiments over nearly a decade, we thought that it was fair to include all the samples for analysis. Even if this sample in the CD34^+^ group was excluded in the subsequent analysis of the three groups, the VIP scoring and trends for TCA, glycolysis/gluconeogenesis, and ketone body production were unchanged. Second, the brain samples used during metabolomic analysis and MS imaging were from different cohorts of mice because the tissue samples needed to be extracted for metabolomic LC–MS analyses. However, the combination of MS metabolomic analysis and MS imaging techniques and the correlation of their results was the rationale of our current study. For future treatment or cell therapies, such methodologies may contribute to a better understanding of the changes that occur during ischemia and the mechanism of functional improvement.

## Materials and methods

### Neonatal stroke model

All experiments were conducted following protocols approved by the Animal Care and Use Committee at the National Cerebral and Cardiovascular Center, which were in accordance with the National Institutes of Health Guide for the Care and Use of Laboratory Animals. Male and female CB17 (CB-17/Icr-+/+Jcl) mouse pups were used for the experiments (*n* = 37). Postnatal day 12 (P12) pups, which are thought to be equivalent to full-term human newborns at P0^41,42^, were randomly divided into a no-surgery control group and MCAO groups. The pups were subjected to permanent MCAO as described previously^5^. Under isoflurane anesthesia (4.0% for induction and 1.5–2.0% for maintenance), a hole was made in the left temporal bone. The left middle cerebral artery (MCA) was electrocauterized and disconnected distal to its crossing with the olfactory tract. After the insult, the mice were observed for bleeding, awakened in a 32 °C infant warmer and returned to their dams. All mice tolerated the MCAO procedure, and there was no surgical mortality.

### Administration of cells after MCAO

Human umbilical-cord-blood-derived CD34^+^ (CD34^+^) cells were purchased from Lonza Inc. (Walkersville, MD). Before use, the cells were thawed and processed according to the producer’s instructions. The cell purity was > 95%, and the viability of the cells was > 90%. Human umbilical cord tissues were collected from women who underwent cesarean section after informed consent was obtained. Human umbilical-cord-derived mesenchymal stem cells (hUC-MSCs: MSCs) were isolated and expanded as described by Mori et al.^43^, followed by validation of their differentiation potential and cell surface molecules^44^. Then, the cells were frozen for storage and thawed just before use.

Forty-eight hours after MCAO insult, the cells were intravenously administered as described previously^45^. Briefly, a skin incision was made while the mouse was under isoflurane anesthesia, and the left femoral vein was exposed. CD34^+^ cells (1 × 10^5^ cells), MSCs (1 × 10^5^ cells) or an equivalent volume (60 μL) of the cryoprotectant STEM-CELLBANKER (ZENOAQ Resource Co., Ltd., Fukushima, Japan) was carefully infused into the femoral vein over 1 min using a 35-gauge needle.

### Sample collection

At P15, which was 24 h after cell administration, samples were obtained. For metabolomic analysis, the mice were decapitated, and the heads were immediately submerged in ice-cold PBS to minimize postmortem changes. Then, the brains were dissected from the skull on dry ice, and 20–30 mg of coronal sections from the left hemisphere, located 4–6 mm from the frontal tip, were stored at − 80 °C until required for analysis (*n* = 5 per group). For MS imaging, another cohort of mice on the same postnatal day were anesthetized under isoflurane, and their scalps were opened. They were immediately submerged in liquid nitrogen and subsequently decapitated, and the frozen brains were dissected from the skull and placed on dry ice. Fifteen-millimeter-thick coronal sections of the whole brain containing the hippocampus and thalamus were obtained. The sections were mounted on glass slides (25 × 75 mm, S8902, Sigma-Aldrich, St. Louis, MO). The adhered tissue sections were then stored at − 80 °C.

### Metabolomic analysis

Explorative analysis of 900 metabolites was performed using capillary electrophoresis time-of-flight mass spectrometry (CE-TOF MS) to investigate the influence of MCAO insult and the effect of subsequent cell therapies.

Approximately 30–50 mg of frozen brain tissue was placed in 1500 μL of 50% acetonitrile/Milli-Q water containing internal standards (Solution ID: 304-1002, Human Metabolome Technologies Inc, Tsuruoka, Japan) at 0 °C. The hemispheric samples were homogenized two times at 1500 rpm for 120 s using a tissue homogenizer (Micro Smash MS100R, Tomy Digital Biology Co., Ltd., Tokyo, Japan), and then the homogenate was centrifuged at 2300*g* (4 °C) for 5 min. Subsequently, 400 μL of the upper aqueous layer was filtered through a Millipore 5 kDa cutoff filter by centrifugation at 9100*g* and 4 °C for 120 min to remove proteins. The filtrate was concentrated through centrifugation and resuspended in 50 μL of Milli-Q water for CE-TOF MS analysis. Metabolomic measurements were carried out by a service facility at Human Metabolome Technology Inc., Tsuruoka, Japan. CE-TOF MS experiments were performed using an Agilent CE-TOF MS system (Agilent Technologies, Santa Clara, CA). Cationic metabolites were analyzed with a fused-silica capillary (50 mm i.d., 80 cm total length) and a cation buffer solution (Human Metabolome Technologies Inc.) as the electrolyte. The applied CE voltage was set at 27 kV. Electrospray ionization-mass spectrometry (ESI–MS) was conducted in positive ion mode, and the capillary voltage was set at 4000 V. The spectrometer was scanned from *m*/*z* 50 to 1000. Anionic metabolites were analyzed under basically the same conditions used for the cation analysis with slight modifications: an anion buffer solution (Human Metabolome Technologies Inc) was used as the electrolyte, the CE voltage was set to 30 kV, and the ESI–MS capillary voltage was set to 3500 V in negative ion mode. The ESI conditions were as follows: nebulizer pressure, 5 psi for cations (Method: TOF-C03), 8 psi for anions (Method: TOF-A05); dry gas, nitrogen; flow rate, 7 L/min; and temperature, 300 °C. Metabolites in the samples were identified by comparing the migration time and *m*/*z* ratio with those of authentic standards (Solution ID: H3304-1031, H3304-1032, H3304-1034 and H3304-1036, Human Metabolome Technologies Inc). The identified metabolites were quantified by comparing their peak areas with those of authentic standards using Chem Station software (Agilent Technologies, Santa Clara, CA).

### MS imaging of brain sections

The brain sections mounted on the glass slides were coated with 2,5-dihydroxybenzoic acid (DHB) using an ImagePrep tissue imaging matrix sprayer (Bruker Daltonics, Bremen, Germany). MALDI-MS imaging was performed on a 7 T SolariX FT-ICR MS (Bruker Daltonics, Bremen, Germany) in positive ion mode. A mass range of *m*/*z* 71–1000 was employed with a time domain for acquisition of 2 M (duration 0.699 s), providing an estimated resolving power of 94,000 at *m*/*z* 400. The instrument settings corresponding to "Ion Transfer" were adjusted to maximize the intensity of ions for authentic standards (sodium formate, Sigma-Aldrich) on the low-MS side. Before each batch of analyses, external calibration was performed by sodium formate infusion via an ESI source. Lock mass calibration was performed at m/z 273.039364 (matrix ion^15^) with FT MS control software (Bruker Daltonics, Bremen, Germany). Each spectrum encompassed 500 laser shots: laser focus, medium; laser power, 45%; laser spot, < 60 µm. The spatial resolution was set to 100 µm. Optical images of tissue sections were acquired using an Epson GT-X830 flatbed scanner using a minimum setting of 2400 d.p.i. The acquired mass spectrometry data were analyzed using FlexImaging 4.1 software (Bruker Daltonics, Bremen, Germany) and were normalized to *m*/*z* 273 (DHB [M + H]^+^). Hierarchical cluster analysis was conducted using a peak tolerance of mass errors as follows: for glucose [M + Na]^+^, *m*/*z* = 234.0526 ± 5 ppm; for carnitine [M + H]^+^, *m*/*z* = 162.1125 ± 4 ppm; for glutamate [M + Na]^+^, *m*/*z* = 170.0424 ± 4 ppm; for glutamate [M + H]^+^, *m*/*z* = 148.0604 ± 4 ppm; for glutathione [M + Na]^+^, *m*/*z* = 330.0730 ± 6 ppm; and for glutathione [M + H]^+^, *m*/*z* = 308.0911 ± 6 ppm. Individual spectra were analyzed using Data Analysis 4.4 software, with peak lists generated using a signal-to-noise threshold = 0.05% of the base peak-height threshold.

### Data analysis for relative accumulation in the regions of interest (ROIs)

For quantification purposes, the specific regions of interest (ROIs) were manually defined using FlexImaging software. The infarct area in this model was clearly demarcated 48 h after MCAO, and the peri-infarct area was defined as the 0.4 mm-thick border adjacent to the infarct area. The remaining area was named the non-infarct area. The average spectrum was generated in Data Analysis software for each specific ROI. The average signal intensities expressed in *m*/*z* were normalized with the intensity of the respective matrix signal and then compared.

### Statistical analysis

For the analysis of the metabolites, principal component analysis, partial least-squares discriminant analysis (PLS-DA), heat map generation and metabolite set enrichment analysis were performed using the freely available software program MetaboAnalyst 4 (https://www.metaboanalyst.ca/). Metabolites with more than 20% missing data were eliminated from subsequent statistical analysis. The concentrations of metabolites are expressed as the mean ± the S.E.M. and analyzed by one-way ANOVA with Dunn’s post hoc tests. Calculated values of relative intensity in MS imaging were assessed using a Kruskal–Wallis test followed by Dunn’s test using JMP 12.2.0 software (SAS Institute, Cary, NC, USA). Results with *p* values < 0.05 were considered statistically significant.

## Supplementary Information

Supplementary Table.

Supplementary Figure S1.

Supplementary Figure S2.

Supplementary Dataset.

## Data Availability

The data that support the findings of this study are available from the corresponding author upon reasonable request.
