# Supplementary material for: Metabolomic analysis and mass spectrometry imaging after neonatal stroke and cell therapies in mouse brains
Source: Sci Rep. 2020 Dec 14;10:21881. doi: 10.1038/s41598-020-78930-x (PMC7736587; doi:10.1038/s41598-020-78930-x)
Supplement: Supplementary file 1 — Supplementary Table. [file 41598_2020_78930_MOESM1_ESM.pdf]

Principal component analysis

	KEGG ID	PC1 vector	Factor loading	*p* value		KEGG ID	PC1 vector	Factor loading	*p* value
Negative correlation with PC1					Positive correlation with PC1				
Isethionic acid	C05123	-0.125	0.967	1.89E-08	Ile	C00407	0.122	0.942	4.62E-07
Asn	C00152	-0.125	0.965	2.43E-08	Putrescine	C00134	0.121	0.938	6.98E-07
N-Acetylaspartic acid	C01042	-0.124	0.960	5.45E-08	2'-Deoxycytidine	C00881	0.121	0.935	9.16E-07
XA0065	No ID	-0.123	0.955	1.14E-07	Leu	C00123	0.120	0.933	1.15E-06
N-Acetylglutamic acid	C00624	-0.122	0.942	5.08E-07	Val	C00183	0.120	0.930	1.43E-06
N-Acetylneuraminic acid	C00270	-0.121	0.938	7.43E-07	Ornithine	C00077	0.119	0.920	3.19E-06
Succinic acid	C00042	-0.118	0.911	5.95E-06	Lys	C00047	0.117	0.908	7.22E-06
Creatine	C00300	-0.117	0.903	9.71E-06	Cystathionine	C00542	0.117	0.907	7.62E-06
Glu	C00025	-0.114	0.884	2.67E-05	XC0132	No ID	0.117	0.903	9.84E-06
GDP-glucose	C00394	-0.114	0.882	2.99E-05	Phe	C00079	0.115	0.894	1.65E-05
UDP-glucose	C00029	-0.113	0.874	4.33E-05	Pro	C00148	0.114	0.880	3.28E-05
Glutathione GSSG_divalent	C00127	-0.110	0.852	1.10E-04	ADMA	C03626	0.112	0.869	5.58E-05
Ethanolamine phosphate	C00346	-0.110	0.850	1.17E-04	Betaine	C00719	0.110	0.853	1.04E-04

CDP-choline	C00307	-0.109	0.841	1.63E-04	Guanidoacetic acid	C00581	0.110	0.852	1.09E-04
NADP	C00006	-0.108	0.837	1.86E-04	Trp	C00078	0.109	0.841	1.67E-04
1-Aminocyclopropane-1-carboxylic acid	C01234	-0.108	0.833	2.16E-04	Citrulline	C00327	0.108	0.835	2.02E-04
S-Adenosylmethionine	C00019	-0.107	0.828	2.59E-04	Arg	C00062	0.108	0.835	2.06E-04
UDP-N-acetylgalactosamine	C00203	-0.106	0.822	3.05E-04	Thr	C00188	0.107	0.829	2.44E-04
Saccharopine	C00449	-0.105	0.812	4.25E-04	Met	C00073	0.106	0.820	3.34E-04
Asp	C00049	-0.104	0.808	4.69E-04	myo-Inositol 1-phosphate	C01177	0.106	0.818	3.52E-04
XA0033	No ID	-0.104	0.805	5.07E-04	His	C00135	0.101	0.783	9.33E-04
CMP-N-acetylneuraminate	C00128	-0.102	0.787	8.37E-04	N8-Acetylspermidine	C01029	0.098	0.763	1.52E-03
Malic acid	C00149	-0.100	0.774	1.16E-03	N,N-Dimethylglycine	C01026	0.096	0.746	2.20E-03
GDP	C00035	-0.099	0.771	1.25E-03	Isobutyrylcarnitine	No ID	0.096	0.745	2.25E-03
Fumaric acid	C00122	-0.099	0.767	1.36E-03	Hypotaurine	C00519	0.096	0.741	2.45E-03
XA0019	No ID	-0.098	0.760	1.63E-03	O-Acetylcarnitine	C02571	0.095	0.739	2.51E-03
g-Butyrobetaine	C01181	-0.098	0.758	1.69E-03					
XC0137	No ID	-0.097	0.749	2.03E-03					
AMP	C00020	-0.096	0.746	2.20E-03					
Pyridoxamine 5'-phosphate	C00647	-0.096	0.743	2.33E-03					

N-Acetylglucosamine 1-phosphate	C04256	-0.096	0.742	2.40E-03
Phosphorylcholine	C00588	-0.092	0.715	4.06E-03
