# Supplementary figures and images for: Metabolomic analysis and mass spectrometry imaging after neonatal stroke and cell therapies in mouse brains
Source: Sci Rep. 2020 Dec 14;10:21881. doi: 10.1038/s41598-020-78930-x (PMC7736587; doi:10.1038/s41598-020-78930-x)

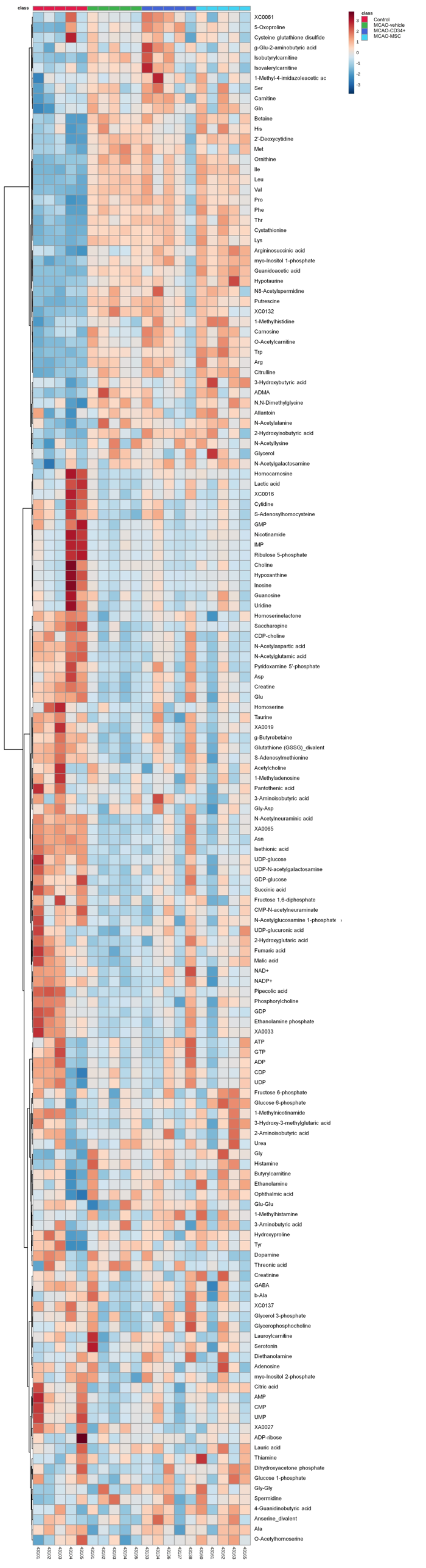

Supplement: Supplementary file 2 — Supplementary Figure S1. [file 41598_2020_78930_MOESM2_ESM.jpg]

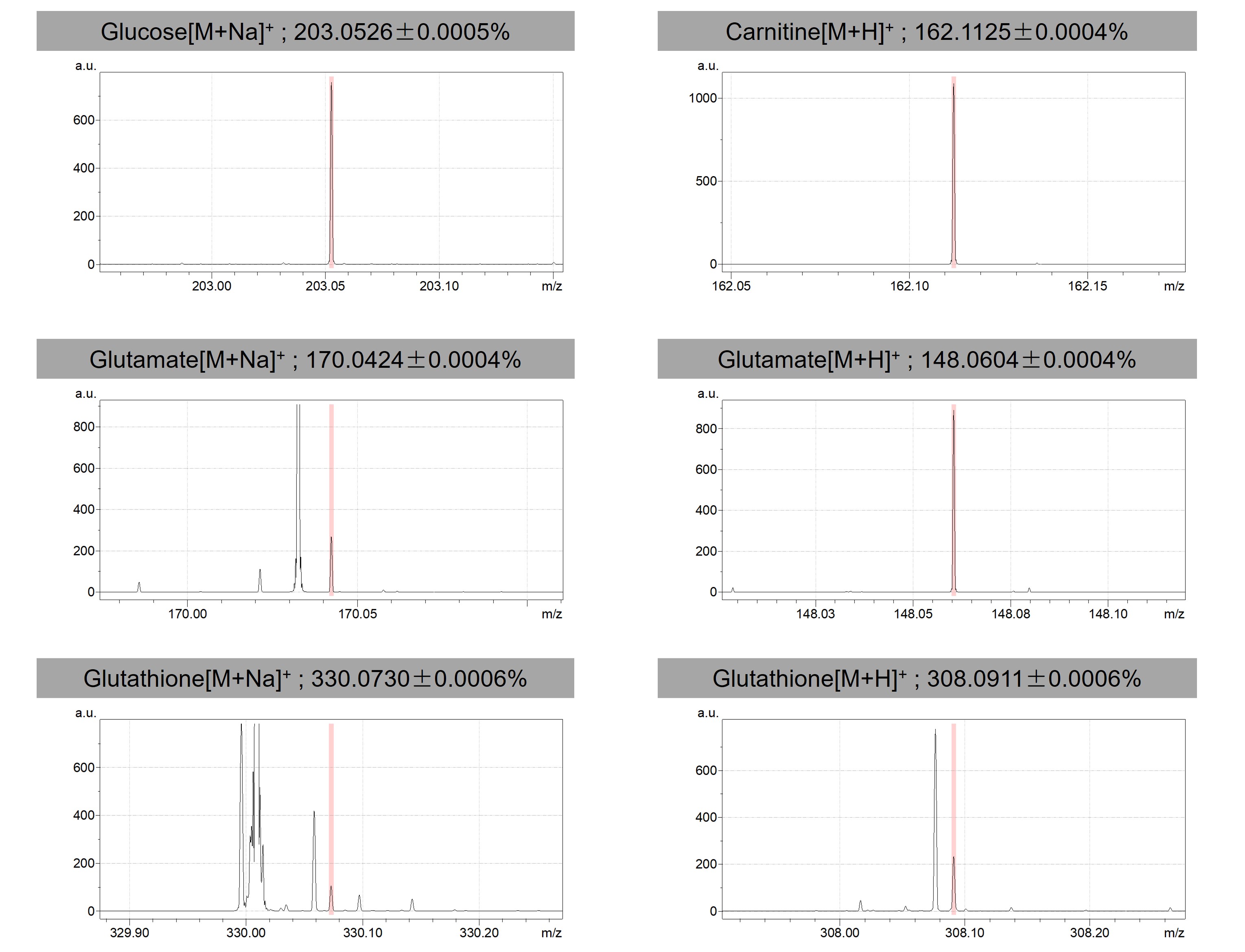

Supplement: Supplementary file 3 — Supplementary Figure S2. [file 41598_2020_78930_MOESM3_ESM.jpg]
